# Ambient particulate matter and biomass burning: an ecological time series study of respiratory and cardiovascular hospital visits in northern Thailand

**DOI:** 10.1186/s12940-020-00629-3

**Published:** 2020-07-03

**Authors:** W. Mueller, M. Loh, S. Vardoulakis, H. J. Johnston, S. Steinle, N. Precha, W. Kliengchuay, K. Tantrakarnapa, J. W. Cherrie

**Affiliations:** 1 Institute of Occupational Medicine, Edinburgh, EH14 4AP UK; 2 Australian National University, Canberra, Australia; 3 Heriot Watt University, School of Engineering and Physical Sciences, Institute of Biological Chemistry, Biophysics and Bioengineering, Riccarton, Edinburgh, EH14 4AS UK; 4 Mahidol University, Bangkok, Thailand; 5 Walailak University, Nakhon Si Thammarat, Thailand

**Keywords:** Particulate matter, Biomass burning, Thailand, Hospital visits, Time series, Ambient air pollution

## Abstract

**Background:**

Exposure to particulate matter (PM) emitted from biomass burning is an increasing concern, particularly in Southeast Asia. It is not yet clear how the source of PM influences the risk of an adverse health outcome. The objective of this study was to quantify and compare health risks of PM from biomass burning and non-biomass burning sources in northern Thailand.

**Methods:**

We collected ambient air pollutant data (PM with a diameter of < 10 μm [PM_10_], PM_2.5_, Carbon Monoxide [CO], Ozone [O_3_], and Nitrogen Dioxide [NO_2_]) from ground-based monitors and daily outpatient hospital visits in Thailand during 2014–2017. Outpatient data included chronic lower respiratory disease (CLRD), ischaemic heart disease (IHD), and cerebrovascular disease (CBVD). We performed an ecological time series analysis to evaluate the association between daily air pollutants and outpatient visits. We used the 90th and 95th percentiles of PM_10_ concentrations to determine days of exposure to PM predominantly from biomass burning.

**Results:**

There was significant intra annual variation in PM_10_ levels, with the highest concentrations occurring during March, coinciding with peak biomass burning. Incidence Rate Ratios (IRRs) between daily PM_10_ and outpatient visits were elevated most on the same day as exposure for CLRD = 1.020 (95% CI: 1.012 to 1.028) and CBVD = 1.020 (95% CI: 1.004 to 1.035), with no association with IHD = 0.994 (95% CI: 0.974 to 1.014). Adjusting for CO tended to increase effect estimates. We did not find evidence of an exposure response relationship with levels of PM_10_ on days of biomass burning.

**Conclusions:**

We found same-day exposures of PM_10_ to be associated with certain respiratory and cardiovascular outpatient visits. We advise implementing measures to reduce population exposures to PM wherever possible, and to improve understanding of health effects associated with burning specific types of biomass in areas where such large-scale activities occur.

## Introduction

Ambient air pollution, and most notably particulate matter (PM), causes significant harm on a global scale, including over 4 million attributable deaths [1] and 5 million asthma emergency department visits [2] annually. Sources of PM can be both anthropogenic (e.g., traffic, industry) and natural (e.g., dust, sea salt) [3]. One significant contribution of PM emission is biomass burning of both natural and anthropogenic origin, including wildfires, agricultural residue burning, land clearing, and domestic fuel burning [4]. Historically, most research on the health risks of PM has been documented in urban areas where PM emissions are mainly derived from traffic, domestic sources, and industry [5]. Regional, as well as long-range, atmospheric transport also affects PM concentrations in urban areas [6]. While a very active research area, there is, at present, no scientific consensus on differentiated health risks of PM from different sources [7].

Most PM time series studies have focused on mortality outcomes [8], and many have also demonstrated the harmful effect of elevated ambient PM concentrations on hospital admissions, particularly for cardiovascular and respiratory diseases. A meta-analysis indicated about a 1% increase in such admissions for each 10 μg/m^3^ rise in ambient PM_2.5_ [9]. More recent studies have investigated the effects of PM on health in other geographical regions and from more diverse PM sources, including biomass burning. A review of the health effects of wildfire smoke identified consistent evidence of respiratory morbidity, though less clear effects on cardiovascular health [10]. An examination of both epidemiological and toxicological studies concluded that it was not yet clear if urban (traffic) and biomass-derived PM entail differential health hazards [11].

Although satellite imagery indicates a global decline in the number of active biomass fires, parts of Asia, due to agricultural intensification and crop burning, have undergone increasing fire activity [12]. The population of the upper north of Thailand is subject to annual smoke haze events during the dry season, mainly January to April and typically culminating in March, from biomass burning activities in both Thailand and neighbouring countries (e.g., Burma and Indonesia [13]). Biomass burning in agricultural fields is practised to remove residues after the harvest and to manage weeds, while in forests it can contribute to agricultural clearing and assist with the collection of food products [14]. Kliengchuay et al. [15] identified that nearly 10% of daily PM_10_ concentrations in Mae Hong Son Province, Thailand were in excess of the 120 μg/m^3^ daily PM_10_ standard set by the Thailand Pollution Control Department (PCD); such exposure has also been linked to additional pneumonia cases in the region [16]. The Government has attempted to prevent haze by patrolling and extinguishing fires during the critical period, with the involvement of local communities, but this task faces myriad practical obstacles [17].

We have undertaken a time-series analysis to investigate the association of daily PM levels, from biomass burning and other sources, with respiratory and cardiovascular hospital outpatient visits in northern Thailand. We hypothesised that there would be no difference in the health effects associated with PM between biomass burning and other sources. This study is part of the larger research project to study the effects of air pollution in Thailand: Thailand Air Pollution Health Impact Assessment (TAPHIA).

## Methods

### Study setting

**Fig. 1:**
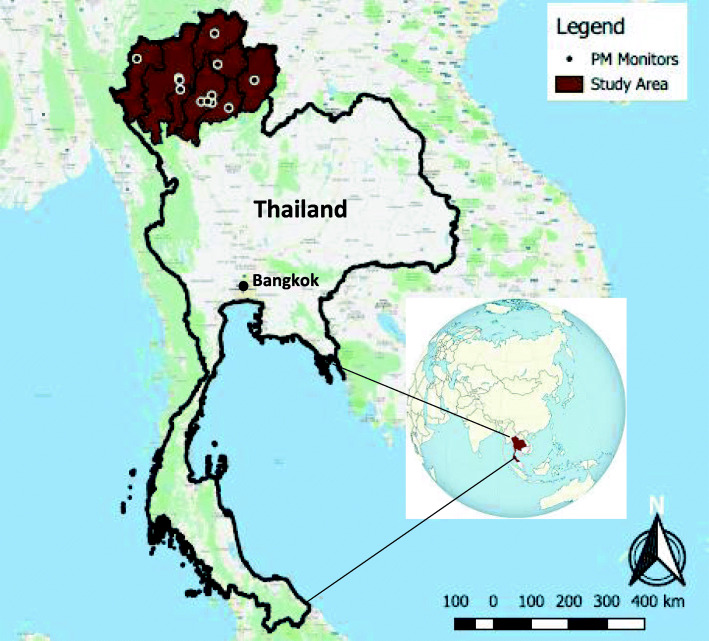
Map depicting the study area in the surrounding region and continent

Thailand, situated in Southeast Asia, has an overall population of nearly 70 million [18] and is organised into 77 provinces. Thailand’s economy has undergone a transition from agricultural to manufacturing and services-based, with the total forest cover reduced from over 50% of land area in the 1960s to about one third in the 2000s; while now stabilised on a national level, deforestation has continued in northern Thailand [19]. The burning of crop residues in Thailand is estimated to release 143,000 t of PM_10_ annually, as well as large amounts of gases and organic compounds, most significantly from rice straw burning [20]. For the present study, we focussed on eight provinces with permanent air quality monitors in the upper north region (i.e., the study area; Fig. 1), including Chiang Mai, Lamphun, Lampang, Phrae, Nan, Phayao, Chiang Rai, and Mae Hong Son, with a combined population of 5.4 million.

### Exposure data

The PCD manages 63 permanent ground stations to monitor ambient levels of air pollutants across Thailand. We collected hourly data from all monitors over the period 1996 to 2017, including the pollutants PM_10_, PM_2.5_, carbon monoxide (CO), nitrogen dioxide (NO_2_), ozone (O_3_), as well as temperature and relative humidity. We identified all monitors located in the study area and selected those with < 25% missing data during 2014–2017 (i.e., the entire study period) to align with health data (described in the following section). We primarily examined the effects of PM, for which we focussed on PM_10_, as sufficient PM_2.5_ data were available from only two stations during the study period however, mean daily PM_10_ and PM_2.5_ values were highly correlated (Spearman’s rho = 0.88) at these sites. Each province in the study area contained at least one background air quality monitor to provide an indication of daily PM_10_ levels (*n* = 12, after exclusion of two ‘traffic’ orientated air quality monitors). Mean daily data from each monitor were deemed to be sufficient and included in the analysis if ≥75% of measurements (i.e., 18 h) were available on a given day [21]. For O_3_, maximum daily values of the 8-h rolling average were used, where at least six hours of data were available [22]. Each province was assigned the mean daily value from the monitors within its boundary; the average value was used if more than one monitor provided data on a given day in each province.

### Health data

Since 2002, the Thai government has funded universal health coverage for its citizenry. This policy has undergone various changes since its inception, including the expansion of access in 2012 to both public and private hospitals for emergency medical services [23]. We obtained individual records of all daily outpatient hospital visits (i.e., emergency and scheduled) from the Thailand Ministry of Public Health for the years 2010 to 2017. All outpatient records were anonymised and included the date, province, sex, age, and reason for visit based on the International Classification of Diseases (ICD-10); the records did not include the actual healthcare facility or nature of visit (i.e., emergency or scheduled). We collected outpatient data on chronic lower respiratory disease (CLRD) visits (ICD-10: J40-J47), ischaemic heart disease (IHD) (ICD-10: I20-I25), and cerebrovascular disease (CBVD) (ICD-10: I60-I69). We excluded for analysis the years 2010–2013, due to reporting limitations associated with the aforementioned changes in universal health coverage; therefore, the study period was 2014–2017.

### Statistical analysis

We performed an ecological time series analysis to examine the association between concentrations of ambient air pollutants and the above respiratory and cardiovascular hospital visits on a daily basis. We employed generalised linear models using a Poisson regression to generate incidence rate ratios (IRR). To adjust for long-term trends and seasonality, we included cubic splines with seven knots per year, and accounted for increased variance in the outcome data by scaling standard errors using the square root of the Pearson Chi-squared-based dispersion [24]. We also controlled for age group (respiratory visits = 0–14, 15–64, 65+ years; cardiovascular visits = 0–64, 65+ years), day of the week, sex, province, and mean daily temperature and relative humidity. Outpatient visits with missing or unreasonable ages (i.e., > 110 years old) or missing gender were omitted from analysis (*n* = 720). We included zero admissions where no daily outpatient records were indicated for a given province, sex, and age group category. We ran models separately for respiratory and cardiovascular (i.e., IHD, CBVD, and IHD + CBVD) visits as the dependent variable.

We examined the effect of mean concentrations of air pollutants on the same day as visits, as well as through the use of a lag for concentrations on the preceding 1–5 days, both individually and cumulatively. We included in the model a binary indicator to identify when biomass burning during January to April made an important contribution to overall PM exposure, according to the 95th percentile of PM_10_ over the entire study area during the full study period (i.e., 1 January 2014 to 31 December 2017), which was 109.6 μg/m^3^. Previous research in the study area indicated PM levels during March and April were moderately to strongly correlated to ambient markers of biomass burning [25] and the number of active fires [26]. We specified an interaction term to examine any differentiation in hospital visits between PM_10_ exposure during burning and non-burning days [27]. Although this method would elucidate any possible differential risks, it could not disentangle any attenuation from PM source or concentration level (i.e. a flattening of the exposure response curve at higher concentrations [28]). We ran all models first with PM_10_, then, if Spearman correlations between PM_10_ and the gaseous pollutants were < 0.7, adjusted for a second pollutant [22]. Model outputs represent IRRs per 10 μg/m^3^ increase in PM_10_. In addition, we examined effects of PM_10_ separately by age (< 65/≥65 years) and sex, and performed a sensitivity analysis to reduce the biomass burning threshold to the 90th percentile (87.1 μg/m^3^). All statistical analysis was completed using Stata (v15).

## Results

**Fig. 2:**
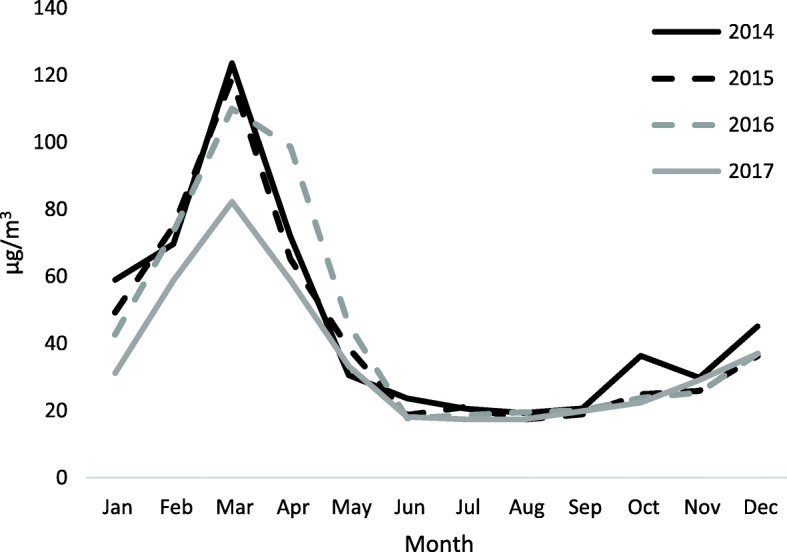
Mean monthly values of PM_10_ during the study period (2014–2017)

**Fig. 3:**
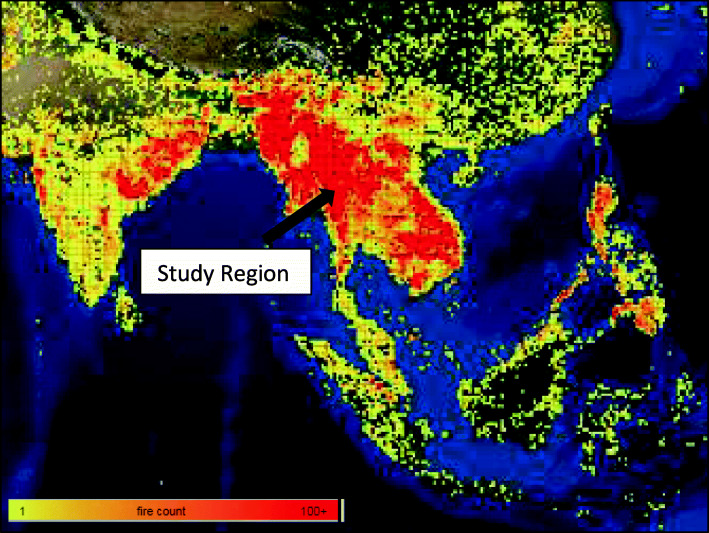
The number of fires in the study region during March 2015 (obtained from NASA’s Fire Information for Resource Management System [FIRMS])

The mean annual PM_10_ and gaseous pollutant concentrations in the study area were lowest in 2017 (Table 1). There was substantial within-year variation in PM_10_ levels, with the highest concentrations consistently experienced during March (Fig. 2), which generally corresponded to the apparent number of fires in the region (Fig. 3; see Fig. S1 for monthly maps of the study region during 2014–2017). There were *n* = 74 and *n* = 147 days across the study period in excess of the 95th and 90th percentiles, respectively, and all but one (in September) occurred during February to April. Spearman correlations between PM_10_ and the gaseous pollutants were moderate to strong: 0.57 (CO), 0.71 (NO_2_), and 0.82 (O_3_) (Table 2). Daily PM_10_ values across the different sites on the same date were strongly correlated (≥0.7). Since the correlation of PM_10_ with O_3_ and NO_2_ were in excess of 0.70, we did not adjust for these pollutants, but we incorporated CO in separate two-pollutant models. Overall, there were 53,694 CLRD, 7752 IHD, and 14,228 CBVD visits over the 1461-day study period (Table 3).

**Table 1:** The mean annual concentrations of pollutants in the study period during 2014–2017

	PM_10_^1^	NO_2_^2^	O_3_^2^	CO^3^
2014	45.6	7.2	24.2	0.59
2015	45.4	8.5	27.6	0.65
2016	44.7	7.7	24.9	0.55
2017	35.5	6.4	21.9	0.54

^1^μg/m^3^

^2^ppb

^3^ppm

**Table 2:** Spearman correlations of daily pollutant concentrations in the study period during 2014–2017

	PM_10_	NO_2_	O_3_	CO
PM_10_	–			
NO_2_	0.71	–		
O_3_	0.82	0.58	–	
CO	0.57	0.50	0.45	–

**Table 3:** Descriptive statistics of daily air pollution and outpatient hospital visits in the study area separated by the burning (January–April) and non-burning (May–December) months during 2014–2017

	Burning				Non-Burning			
	Min	Max	Mean	SD	Min	Max	Mean	SD
Air pollutants								
PM_10_ (μg/m^3^)	5.4	371.1	74.6	42.2	1.6	137.4	26.0	13.7
O_3_ (ppb)	3.6	135.1	59.5	16.9	2.0	92.4	31.7	12.8
CO (ppm)	0	2.9	0.76	0.36	0	1.65	0.48	0.25
NO_2_ (ppb)	0.1	42.4	10.8	6.2	0	29.6	5.7	3.8
Temperature (°C)	7.8	35.8	25.4	4.1	9.3	39.0	26.2	2.8
Relative Humidity (%)	28.0	100.0	64.9	12.1	34.5	100.0	80.7	9.2
Outpatient Hospital Visits (n)								
Chronic Lower Respiratory Disease	6	100	41.0	21.2	3	120	34.7	18.9
< 65 years	0	55	12.6	10.3	0	52	11.1	8.2
≥ 65 years	1	46	15.9	9.0	0	44	12.5	8.0
Male	1	53	19.5	10.5	0	64	17.1	9.6
Female	2	66	21.5	11.7	0	65	17.5	10.3
Ischaemic Heart Disease	0	19	5.1	3.7	0	26	5.4	3.8
< 65 years	0	9	2.3	2.0	0	13	2.3	2.0
≥ 65 years	0	16	2.9	2.0	0	18	3.1	2.5
Male	0	12	2.7	2.2	0	13	2.9	2.4
Female	0	14	2.5	2.1	0	15	2.5	2.1
Cerebrovascular Disease	0	39	9.4	6.5	0	39	9.9	6.6
< 65 years	0	20	4.5	3.4	0	22	4.7	3.6
≥ 65 years	0	19	4.9	3.8	0	21	5.2	3.8
Male	0	18	5.2	3.7	0	21	5.5	3.9
Female	0	22	4.2	3.4	0	18	4.4	3.4

**Fig. 4:**
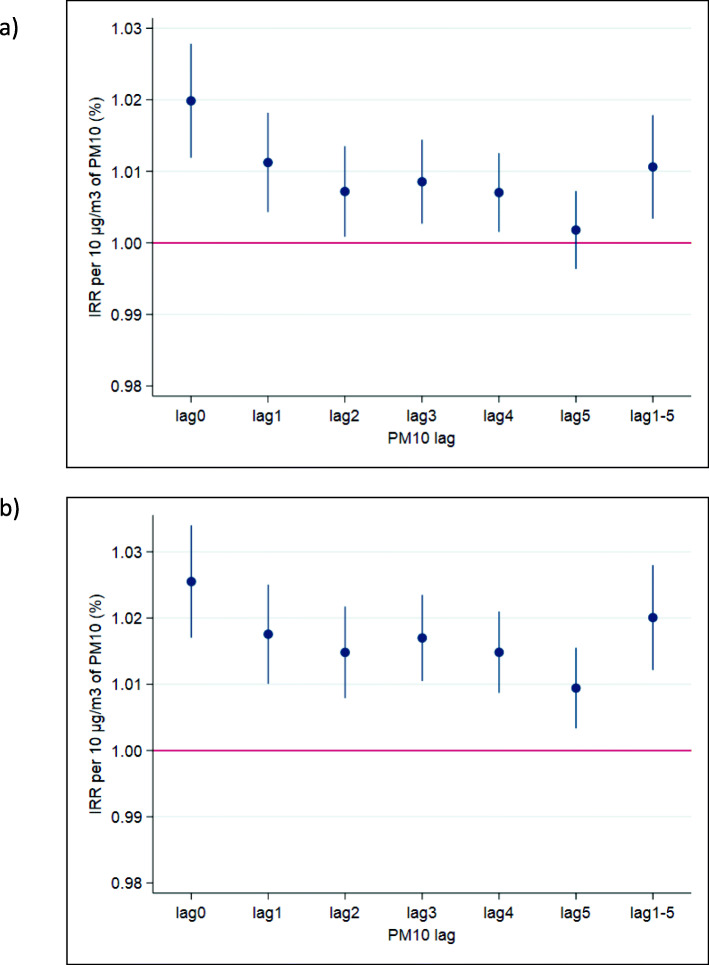
IRRs of CLRD outpatient hospital visits for exposure lags on previous days 1–5 with a) PM_10_ and b) PM_10_ and CO.

The association between daily PM_10_ and CLRD outpatient visits on the same day (i.e., lag0) showed increased IRRs per 10 μg/m^3^ (1.020 [95% CI: 1.012 to 1.028]) with a consistent upward trend (see Fig. S2); however, on days with higher biomass burning-related PM, there was no clear indication of a concentration response association (1.002 [95% CI: 0.993 to 1.012]). Including CO in the model enlarged the IRR for PM_10_ (1.026 [95% CI: 1.017 to 1.035]) (Fig. 4). When stratified by age and sex, risks were increased for both sexes and both < 65 and ≥ 65 years of age for the single pollutant PM_10_ models (see Table 4). The strongest association of PM_10_ exposure with respiratory visits was on the same day, when compared to concentrations on any of the previous five days; IRRs were also elevated with lags 1–4. This trend was consistent when adjusting for the presence of CO (see Fig. 4).

**Table 4:** Incidence Rate Ratios* for PM_10_ exposure on the same day per 10 μg/m^3^, separated by age and sex

	PM_10_	PM_10_ + CO
*Chronic Lower Respiratory Disease*		
Male	**1.012 (1.001 to 1.024)**	**1.017 (1.005 to 1.030)**
Female	**1.028 (1.017 to 1.039)**	**1.034 (1.022 to 1.046)**
< 65 years	**1.021 (1.011 to 1.032)**	**1.029 (1.017 to 1.040)**
≥ 65 years	**1.018 (1.005 to 1.031)**	**1.021 (1.007 to 1.035)**
*Ischaemic Heart Disease*		
Male	0.997 (0.970 to 1.025)	1.001 (0.972 to 1.031)
Female	0.989 (0.961 to 1.018)	0.992 (0.962 to 1.023)
< 65 years	0.973 (0.944 to 1.003)	0.971 (0.940 to 1.002)
≥ 65 years	1.010 (0.983 to 1.037)	1.018 (0.989 to 1.047)
*Cerebrovascular Disease*		
Male	**1.025 (1.004 to 1.046)**	**1.023 (1.001 to 1.045)**
Female	1.013 (0.990 to 1.036)	1.017 (0.993 to 1.042)
< 65 years	1.022 (1.000 to 1.044)	**1.025 (1.002 to 1.049)**
≥ 65 years	1.018 (0.996 to 1.039)	1.016 (0.993 to 1.039)

*Adjusted for season, day of the week, province, mean daily temperature, relative humidity, and days of higher biomass burning-PM (bold results indicate *p* < 0.05)

**Fig. 5:**
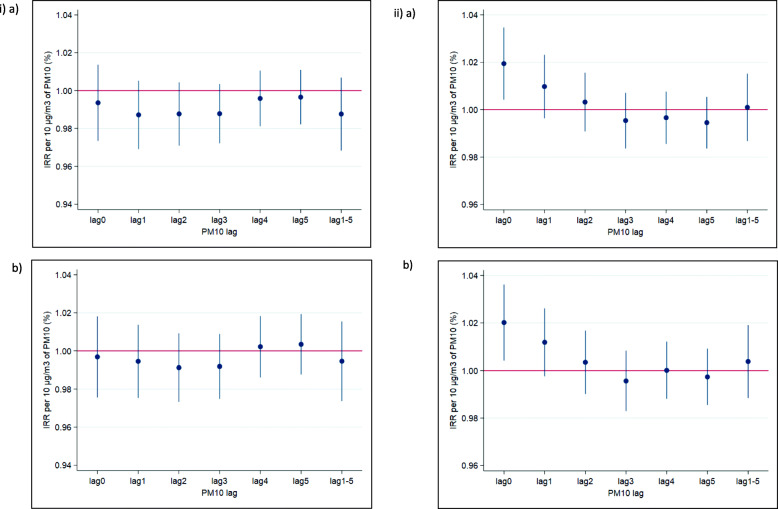
IRRs of i) IHD and ii) CBVD outpatient hospital visits for exposure lags on previous days 1–5 with a) PM_10_ and b) PM_10_ and CO.

There was no apparent association between same-day PM_10_ and IHD visits on non-burning (0.994 [95% CI: 0.974 to 1.014]) or burning days (0.991 [95% CI: 0.964 to 1.019]). By contrast, IRRs for CBVD visits were significantly elevated with PM on non-burning (1.020 [95% CI: 1.004 to 1.035]),but not burning days (0.997 [95% CI: 0.976 to 1.019]); as with the trend for CLRD visits, there was a consistent upward curve with more uncertainty at higher concentrations (Fig. S2). When adjusting for the presence of other pollutants, similar trends were observed as those for CLRD visits: IRRs were slightly increased with CO. When stratified by age and sex, there were no clear increased IHD risks with PM_10_ for both single and multi-pollutant models; coefficients were highest for those aged ≥65 years. Risks with CBVD were elevated for males and for those < 65 years of age, but only when adjusting for CO (Table 4). For CBVD visits, the only significantly elevated risk was observed on the same day of exposure and visit (i.e., lag0), with risk estimates around or below the null for CBVD with exposures on lags 0–5 (see Fig. 5); this pattern held when adjusting for CO.

In the sensitivity analysis at lag0, using the 90th percentile as the threshold for burning slightly reduced the PM_10_ risk estimate with respiratory visits on non-burning days (1.017 [1.007 to 1.026]) and had little effect on that for burning days (1.002 [0.994 to 1.009]). For the cardiovascular outcomes, the lower burning threshold made little difference to the non-burning day IRR for PM_10_ and IHD visits (0.996 [95% CI: 0.973 to 1.020]), but increased the IRR on burning days to 1.015 (95%CI: 0.994 to 1.035). For CBVD visits, the effect of PM_10_ on non-burning days was attenuated to borderline significance (1.017 [95% CI: 0.999 to 1.035]), but there was little difference to the relationship on burning days (0.998 [95% CI: 0.981 to 1.014]).

## Discussion

Our study findings indicate increased outpatient hospital visits for CLRD and CBVD, but not IHD, on the same day as PM_10_ exposures in the upper north of Thailand. These associations were maintained after adjusting for ambient CO concentrations. Evidence of a greater risk with PM_10_ was apparent for different sexes and health outcomes: females (CLRD) and males (CBVD). Although we identified overall risks of PM_10_ with CLRD and CBVD visits when exposure was predominantly from other (i.e., non-biomass burning) sources, we did not identify an exposure-response association for these outcomes on days of higher concentrations with a greater proportion of PM from biomass burning exposure.

We identified more consistent risks for PM_10_ and CLRD visits, which were elevated with both PM_10_ thresholds used to identify biomass burning days, compared to CBVD visits. This result is in agreement with findings from a recent global systematic review on air pollution and cardiorespiratory diseases [29]. The respiratory and cardiovascular systems appear to be most sensitive to the harmful effects of PM [30], and several mechanisms have been identified to explain links to acute events. A host of physiological changes occur with PM exposure that may contribute to exacerbation of existing respiratory disease, including lung and systemic inflammatory responses, and bronchoconstriction [31]. For cardiovascular outcomes, short-term exposure to PM has been associated with such changes as reduced heart rate variability, increased diastolic blood pressure, and enhanced arterial vasoconstriction and blood coagulation, all of which may contribute to acute events [32].

Larger risks on the same day of PM_10_ exposure compared to lagged estimates have also been identified in urban contexts, including all hospital admissions in 218 Chinese cities [33], COPD admissions in Beijing [22], and also specifically with biomass burning: bushfire and respiratory admissions in Australia [34, 35], and haze and respiratory admissions in New Zealand (ages 15–64 years only [36]). Our results with CBVD visits, but not IHD, conflict with findings from Morgan et al. [35], where a relationship with cardiovascular admissions was not identified using any lag. Nevertheless, other studies of non-biomass burning PM have found strongest associations with cardiovascular admissions on the same day as exposure [37–39] (females only). Our results show a more prominent trend for effects only on the same day for cardiovascular (CBVD only) health impacts compared to respiratory outpatient visits, for which there was an apparent increased risk also from prior days’ exposure (until lag4). Mechanistically, for cardiovascular events, the heart and vascular system are susceptible to the negative effects of PM, especially in older people and those with pre-existing heart disease, which can trigger acute events, such as myocardial infarction and stroke [40]. In contrast, although also exacerbated by same day exposures, respiratory events might additionally be precipitated by cumulative exposure over several days before treatment is sought [37].

Our results showed elevated risks across age and sex for CLRD, but not for CBVD visits. When stratifying by sex, females exhibited a higher risk of respiratory visits than males; nevertheless, risks were evident for both sexes, which did not occur for either of the cardiovascular outcomes. Other studies have also found higher coefficients for women and COPD in Beijing [22] and Lanzhou, China [41]. The sex difference in PM_10_ response may, in part, reflect the disparity in smoking prevalence in Thailand among men (40%) and women (2%) [42], which we did not control for in our analysis (similar sex-specific smoking patterns occur in China [43]). Thus, fluctuations in ambient PM may be less important with a larger proportion of smokers, who would receive much higher doses from smoking than from outdoor air. While exposure to (traffic related) PM_10_ has been found not to be related to the development of asthma among smokers (but was in non-smokers) [44], COPD/asthma admissions in London with PM_10_ exposure were found elevated only in current smokers [45]. We found elevated risks for those < 65 years old for CBVD visits when adjusting for CO. In general, research has shown older populations to be more susceptible to PM, though our finding for CBVD is not unique; for example, Su et al. [39] found higher cardiovascular emergency room visits with PM_10_ for those in Beijing aged less than, and not over, 75 years. Ultimately, the < 65 years and ≥ 65 years risk estimates were similar in magnitude, the latter of which perhaps would have gained statistical significance with increased study power; therefore, this result is not necessarily indicative of CBVD risks at younger ages, but at both < 65 and ≥ 65 years old.

Several other time series studies of air pollutants and hospital admissions have been conducted in Thailand. Pongpiachan and Paowa [46] examined gaseous air pollutants and in- and outpatients for respiratory disease in Chiang Mai over 2007–2013 and found the largest positive association with CO (PM was not analysed). Pothirat et al. [47] examined admissions in an open-air facility in Chiang Mai province due to cardiovascular and respiratory diseases over 2016–2017; the dataset used was likely a subset of that in the present study. COPD emergency visits were found to be raised on both the same and subsequent days (i.e., lag0 & lag1), with hospitalisations increased using lag3. No associations were identified with cardiovascular disease, as with IHD visits in the current study. A study in Thailand’s capital, Bangkok, found comparable increases per 10 μg/m^3^ of PM_10_ for total respiratory (1.2%) and cardiovascular (1.0%) admissions as the current study (2.0%), with some similarities in lag trends: significant increases on both lag0 and lag4 for respiratory diseases and on lag0 and lag1 for cardiovascular reasons [48].

In our study, we used the 90th and 95th percentiles of PM_10_ concentrations (i.e., 87.1 μg/m^3^ and 109.6 μg/m^3^, respectively) to identify days of biomass burning exposure, which is slightly lower than the 99 percentile employed by Morgan et al. [35]. A range of other approaches also have been employed to identify days with exposure to burning, or haze, including a doubling of total suspended particulates (mean = 56.9 μg/m^3^ [49]), the extent of discoloration in the sky [36], a threshold of 80 μg/m^3^ to indicate ‘unhealthy’ levels [50], and exposure in the month of March [46]. Other studies of exposure to fires have employed software to track polluted air mass trajectories based on meteorological data (e.g., [51]).

The main corollary of assigning a higher threshold for days with biomass burning is the potential exclusion of some days of actual exposure to predominant biomass burning-derived PM. We did not find evidence that PM_10_ on burning days displayed a clear exposure-response effect, whereas such an association was identified on non-biomass burning days for CLRD and CBVD visits. This result conflicts with some previous studies that have identified biomass burning to either have no difference [27] or be more harmful [52]. If ours is a genuine finding, and not due to constrained statistical power with focussing on higher concentration days, there are two possible interpretations that are not necessarily mutually exclusive.

- First, PM-derived from biomass burning may be less harmful than that from other sources (e.g., traffic-related PM). While not all studies universally demonstrate harmful effects of burning-derived PM in the short-term, particularly for cardiovascular admissions, many studies do implicate such PM with causing adverse health [10].
- Second, it might be the case that at higher PM concentrations, as those used to identify exposure from biomass burning in the present study, the risk begins to subside relative to concentration levels, an effect occurring independently of the source.

Exposure-response trends at higher concentrations have been shown to vary by health outcome and study. Qiu et al. [53] present a flattening of the curve between PM_10_ and respiratory admissions, though no such mitigation was presented by Zhang et al. [54]; however, the latter authors did show such a pattern for cardiovascular admissions. Liu et al. [55] found no indication of curtailed risks at higher concentrations of PM_10_ for ischemic and hemorrhagic stroke hospitalisations. While toxicity studies have indicated potential differences based on the type of biomass, such detail is not widely available for epidemiological data [11]. Another possible explanation for the lack of an exposure-response effect on burning days in our study is that individuals might engage in exposure reduction activities (e.g. staying indoors, wearing a face mask) due to more awareness of poorer air quality during periods of biomass burning [56]. Ultimately, research in other settings with improved assessment of exposure to biomass burning would add clarity to the underlying mechanism and magnitude of risk level.

All of the pollutants in our study (i.e., PM_10_, CO, O_3_, NO_2_) were at least moderately correlated, which presented a challenge to parse out independent effects of each on health. Adjusting for CO increased risk coefficients for both respiratory and cardiovascular outpatient visits; this pattern implies that PM_10_ acts independently of CO. With the high correlations of PM_10_ with O_3_ and NO_2_, we were unable to quantify effects of PM_10_ independently of these pollutants. A review investigating effects of NO_2_ and PM on hospital admissions found NO_2_ to entail independent health effects [57], so unadjusted PM_10_ estimates might also reflect some contribution from NO_2_.

Our study has several strengths, including a population of over 5 million people with over 75,000 outpatient admissions and multiple seasons of biomass burning with which to analyse associations with exposure. Further, our study provides insights on the health effects from exposure to biomass burning in a Southeast Asian context, which is, to date, an underrepresented geographical area [10]. Nonetheless, there are several limitations to address that might have affected our study findings and interpretation. We used outpatient hospital data, but were unable to exclude scheduled visits. As these would have been planned in advance and thus not affected by air pollution levels on the visit date, they may have diluted statistical associations, especially if such visits were cancelled on account of high ambient air pollution levels [58]. For exposure assessment, we relied on a limited number of fixed monitors to capture daily PM_10_ levels. While this source would provide poor spatial resolution, it would be sufficient to reflect temporal variation for time series investigations, given concentrations of the same pollutant at different sites were found to be correlated well in time [59]. As we used levels of PM_10_ to identify biomass burning exposure, and not specific indicators (e.g., levoglucosan [60]), we may have misclassified such exposures. We did not examine the composition of PM_10_ or conduct source apportionment of PM mass, which would have allowed more refined characterization of PM from biomass burning. Using the 90th/95th percentile would restrict exposures to the higher range, reducing the number of events and thus statistical power. In addition, as highlighted previously, we employed a threshold, so we were unable to distinguish potential attenuation by PM source (i.e., biomass burning) or by virtue of higher concentration levels.

## Conclusions

We conducted a time series study examining the effects of PM_10_, including that predominantly from biomass burning, on outpatient hospital visits in the upper north of Thailand. Consistent with current evidence, we found deleterious effects for PM_10_ on respiratory and stroke visits, and we identified the strongest associations on the same day as exposure. Our results regarding the health hazards of exposure to biomass burning PM_10_ should be confirmed in other settings and exposure levels, along with identifying specific types of biomass; PM constituent and source apportionment analyses of health effects should be evaluated more definitively in future research. We advise implementing measures to discourage burning and lessen overall PM exposures in areas where such large-scale activities occur.

## Supplementary information

**Additional file 1: Figure S1.** The total number of fires each month in Southeast Asia during 2014–2017 (obtained from NASA’s Fire Information for Resource Management System [FIRMS]).

**Additional file 2: Figure S2.** The average predicted number of a) Chronic Lower Respiratory Disease (CLRD), b) Ischaemic Heart Disease (IHD), and c) Cerebrovascular Disease (CVD) visits relating to daily mean PM_10_ exposure.

## Data Availability

Access to the data used in this study is governed by the TAPHIA Data Management Plan, which is available at www.TAPHIA-project.org. After the study has ended, anonymised datasets will be made available to other researchers, with the agreement of the TAPHIA researchers, upon request. The TAPHIA research team will decide upon the types of data that may be made available to others, in compliance with the UK Research and Innovation (UKRI) and Thai Research Foundation (TRF) policies on data archiving.

## Abbreviations

CLRD: Chronic Lower Respiratory Disease
CO: Carbon Monoxide
CBVD: Cerebrovascular Disease
ICD: International Classification of Diseases
IHD: Ischaemic Heart Disease
IRR: Incidence Rate Ratio
NASA: National Aeronautics and Space Administration
NO_2_: Nitrogen Dioxide
PCD: Pollution Control Department
PM: Particulate Matter
O_3_: Ozone
VIIRS: Visible Infrared Imaging Radiometer Suite
