# Supplementary material for: Ambient particulate matter and biomass burning: an ecological time series study of respiratory and cardiovascular hospital visits in northern Thailand
Source: Environ Health. 2020 Jul 3;19:77. doi: 10.1186/s12940-020-00629-3 (PMC7333306; doi:10.1186/s12940-020-00629-3)

**Figure S1**. The total number of fires each month in Southeast Asia during 2014-2017 (obtained from NASA’s Fire Information for Resource Management System [FIRMS]).


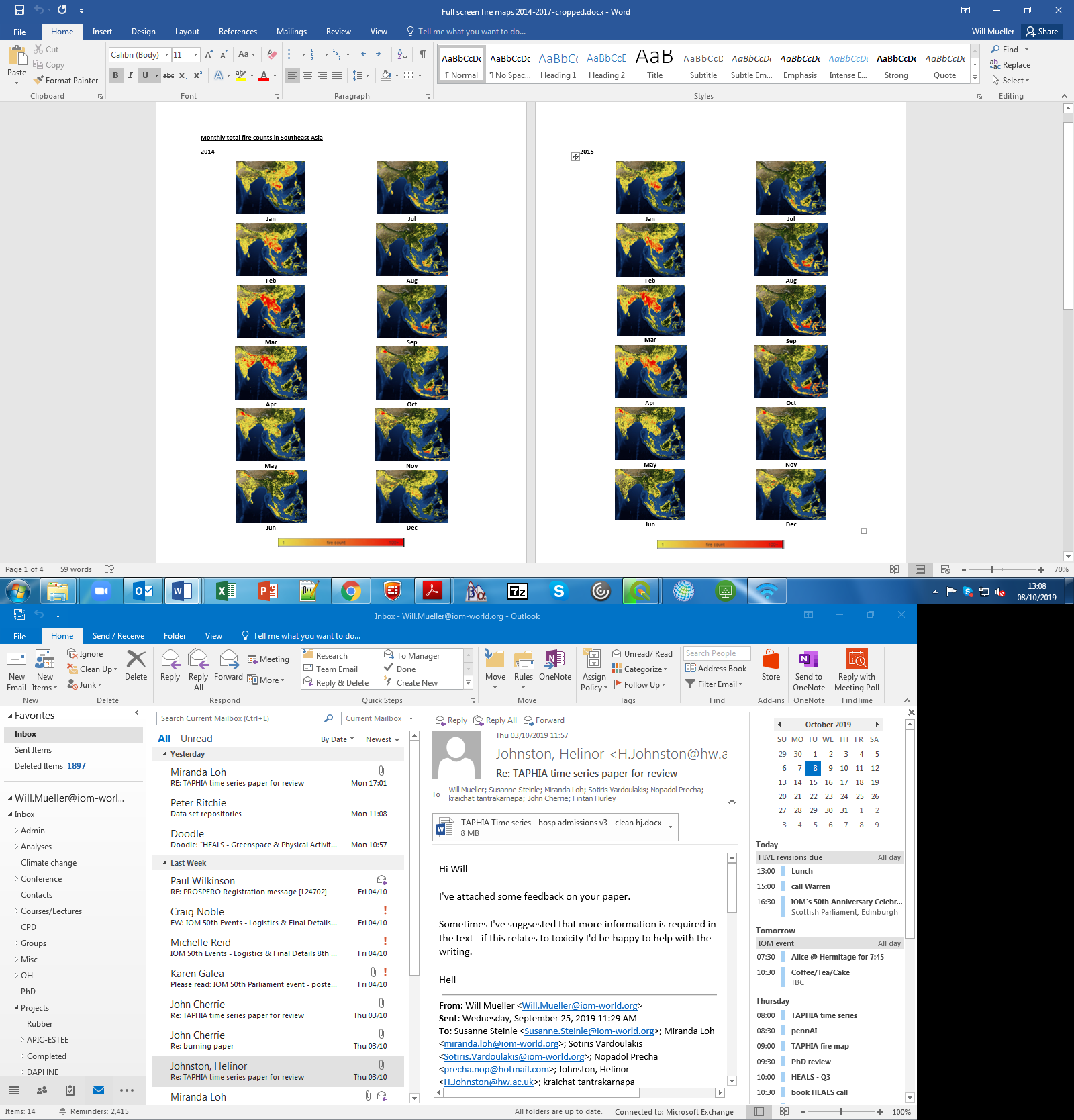


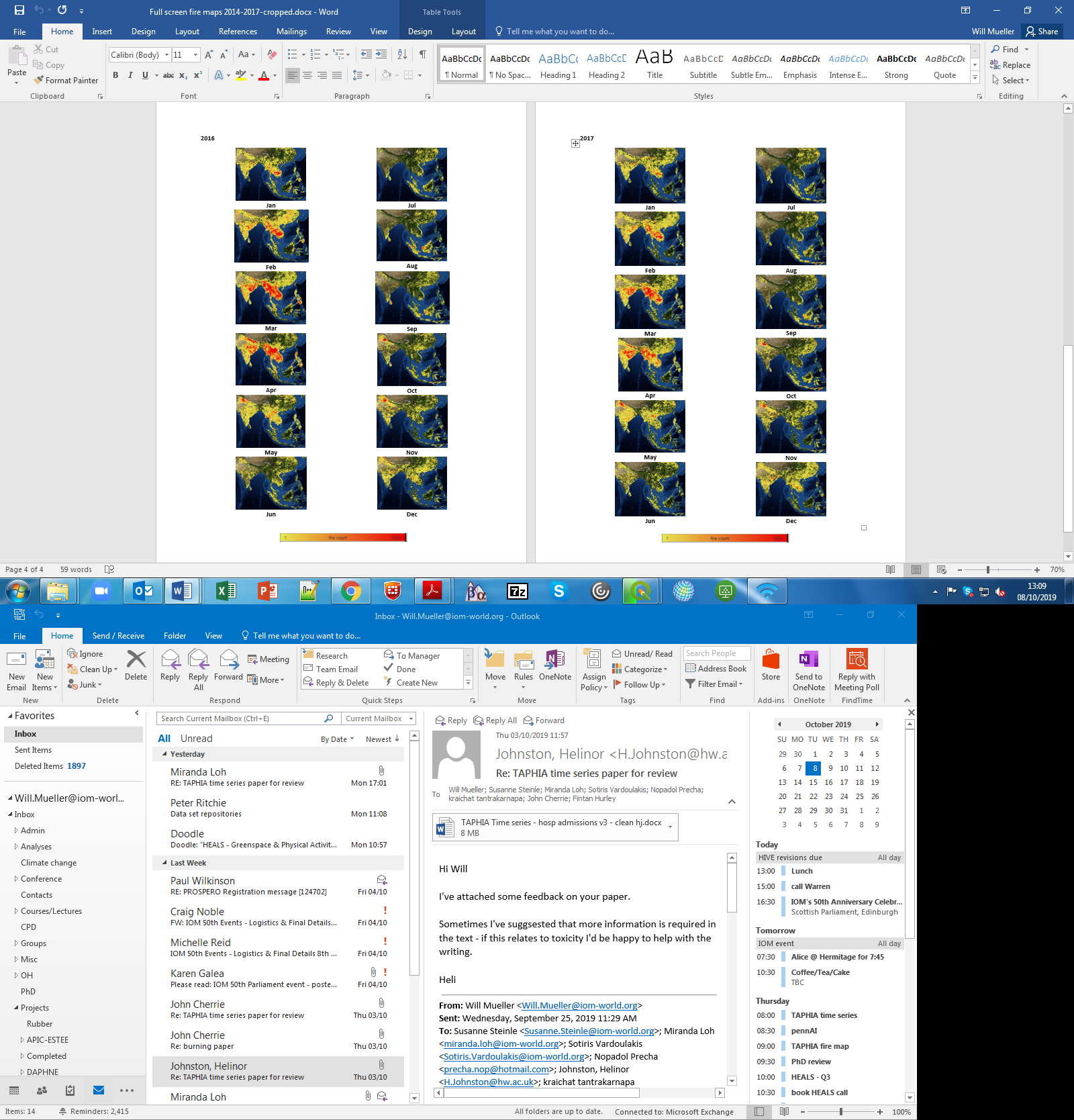


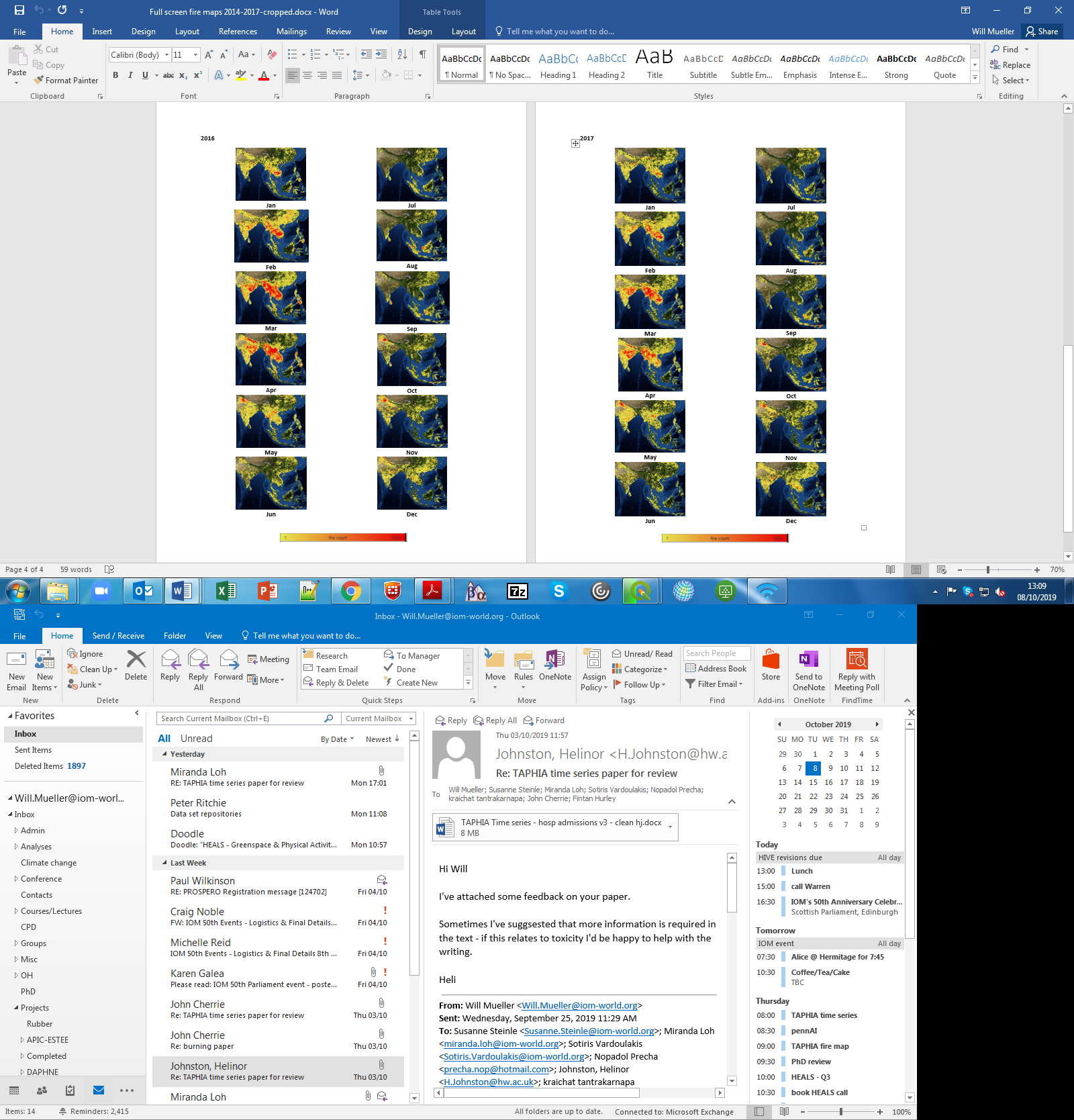

Supplement: Supplementary file 1 — Additional file 1: Figure S1. The total number of fires each month in Southeast Asia during 2014–2017 (obtained from NASA’s Fire Information for Resource Management System [FIRMS]). [file 12940_2020_629_MOESM1_ESM.docx]
