# Supplementary material for: Ambient particulate matter and biomass burning: an ecological time series study of respiratory and cardiovascular hospital visits in northern Thailand
Source: Environ Health. 2020 Jul 3;19:77. doi: 10.1186/s12940-020-00629-3 (PMC7333306; doi:10.1186/s12940-020-00629-3)
Supplement: Supplementary file 2 — Additional file 2: Figure S2. The average predicted number of a) Chronic Lower Respiratory Disease (CLRD), b) Ischaemic Heart Disease (IHD), and c) Cerebrovascular Disease (CVD) visits relating to daily mean PM10 exposure. [file 12940_2020_629_MOESM2_ESM.docx]

**Figure S2**. The average predicted number of a) Chronic Lower Respiratory Disease (CLRD), b) Ischaemic Heart Disease (IHD), and c) Cerebrovascular Disease (CVD) visits relating to daily mean PM_10_ exposure.


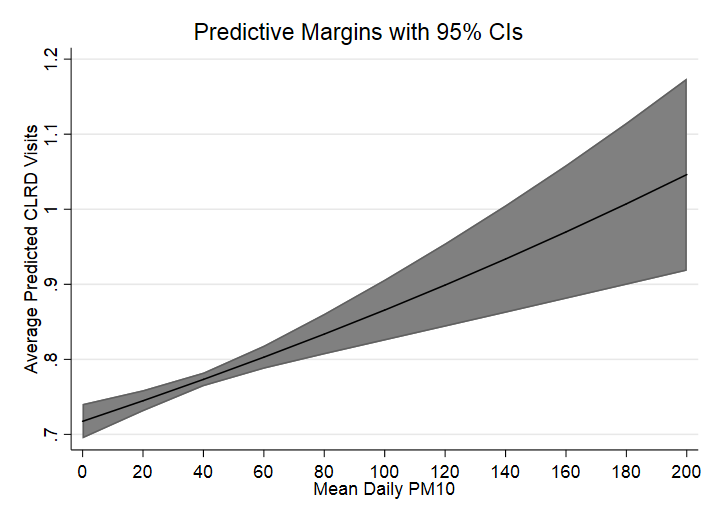
a)


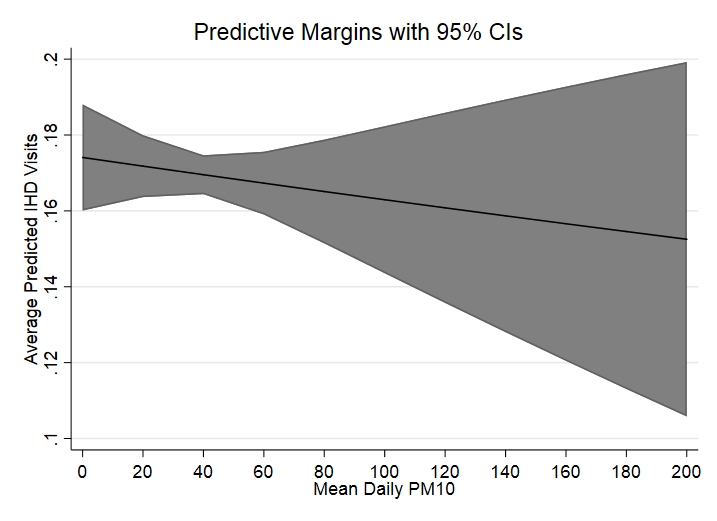
b)


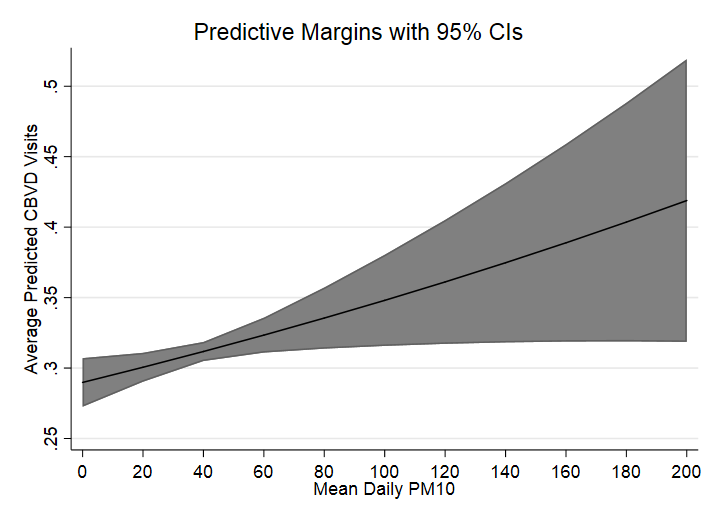
c)
